# Baicalein Reduces Airway Injury in Allergen and IL-13 Induced Airway Inflammation

**DOI:** 10.1371/journal.pone.0062916

**Published:** 2013-04-30

**Authors:** Ulaganathan Mabalirajan, Tanveer Ahmad, Rakhshinda Rehman, Geeta Devi Leishangthem, Amit Kumar Dinda, Anurag Agrawal, Balaram Ghosh, Surendra Kumar Sharma

**Affiliations:** 1 Molecular Immunogenetics Laboratory, and Centre of Excellence for Translational Research in Asthma & Lung Disease, CSIR-Institute of Genomics and Integrative Biology, Mall Road, Delhi, India; 2 Department of Pathology, All India Institute of Medical Sciences, Ansari Nagar, New Delhi, India; 3 Division of Pulmonary, Critical Care and Sleep Medicine, Department of Medicine, All India Institute of Medical Sciences, Ansari Nagar, New Delhi, India; National Jewish Health, United States of America

## Abstract

**Background:**

Baicalein, a bioflavone present in the dry roots of *Scutellaria baicalensis Georgi,* is known to reduce eotaxin production in human fibroblasts. However, there are no reports of its anti-asthma activity or its effect on airway injury.

**Methodology/Principal Findings:**

In a standard experimental asthma model, male Balb/c mice that were sensitized with ovalbumin (OVA), treated with baicalein (10 mg/kg, ip) or a vehicle control, either during (preventive use) or after OVA challenge (therapeutic use). In an alternate model, baicalein was administered to male Balb/c mice which were given either IL-4 or IL-13 intranasally. Features of asthma were determined by estimating airway hyperresponsiveness (AHR), histopathological changes and biochemical assays of key inflammatory molecules. Airway injury was determined with apoptotic assays, transmission electron microscopy and assessing key mitochondrial functions. Baicalein treatment reduced AHR and inflammation in both experimental models. TGF-β_1_, sub-epithelial fibrosis and goblet cell metaplasia, were also reduced. Furthermore, baicalein treatment significantly reduced 12/15-LOX activity, features of mitochondrial dysfunctions, and apoptosis of bronchial epithelia.

**Conclusion/Significance:**

Our findings demonstrate that baicalein can attenuate important features of asthma, possibly through the reduction of airway injury and restoration of mitochondrial function.

## Introduction

Asthma is associated with recruitment of various inflammatory cells such as eosinophils and lymphocytes to the airway. These cells release various reactive free radicals to create an oxidative microenvironment in the airway [1], which injures structural cells of airway such as bronchial epithelial cells. Repeated allergic inflammation leads to irreversible epithelial injury and causes the detachment of ciliated bronchial epithelial cells from the basement membrane. In support of this view, epithelial clumps have been found in sputum (creola bodies) and bronchoalveolar lavage (BAL) of asthmatic patients [2]. While inflammatory cells such as Th_2_ lymphocytes, eosinophils, and mast cells have historically occupied center stage in asthma research, recent reports have highlighted the critical role of epithelial stress in deciding the immune responses within the lung [3]–[7]. Epithelial injury is now considered to be one of the primary pathogenic mechanisms for the development of various features of allergic asthma [8]. Interestingly, restoring mitochondrial function of airway epithelia is sufficient to reduce acute lung injury, indicating the efficiency of mitochondria in maintaining epithelial homeostasis and thus the viability of the lung [9]. We and others have previously demonstrated the association of mitochondrial structural changes in bronchial epithelia with asthma, and the pro-inflammatory effects of mitochondrial dysfunction [10]–[12]. Thus, it is of interest to explore therapeutic agents that can restore mitochondrial function as a means of reducing the deleterious effects of airway epithelial injury.

Baicalein (5, 6, 7-trihydroxy-2-phenyl-4H-1-benzopyran-4-one), a bioflavone component present in the dry roots of *Scutellaria baicalensis Georgi,* has been described in the Chinese Pharmacopoeia as a drug. It possesses a wide range of biological activities such as antiviral, antioxidant, anti-inflammatory, antithrombotic, and anticancer effects [13]. It has been shown to inhibit the activity of the 15-lipoxygenase (15-LOX), which has been implicated in mitochondrial degradation in reticulocytes. It also reduces eotaxin production in cultured human fibroblasts stimulated by the combination of interleukin-4 and TNF-α [14]. Though it has been suggested that baicalein may have anti-allergic effects based on *in-vitro* studies [15], this has not been confirmed *in vivo*. Importantly, baicalein reduces neuronal injury [16], [17], and inhibits retinal epithelial apoptosis induced by hydrogen peroxide [18]. In light of this, we hypothesized that baicalein may have anti-asthma activity.

## Results

### Baicalein Reduces Airway Hyperresponsiveness

**Figure 1:**
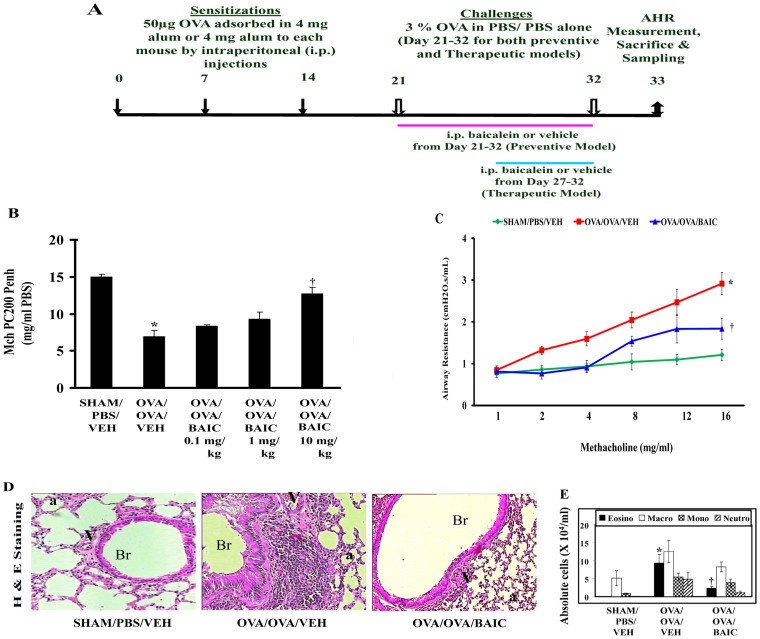
Effect of Baicalein (BAIC) pretreatment on airway hyperresponsiveness (AHR) to Methacholine. A) Male Balb/c mice were sensitized and challenged as shown. AHRs were determined on Day 33. B) In dose titration experiments, single chamber plethysmography results were expressed as MCh PC200 [the partial concentration of methacholine which is required to double the baseline enhanced pause (Penh)]. C) In preventive model, invasive airway mechanics results were expressed as airway resistance. D) Representative photomicrographs of Haematoxylin and Eosin staining were shown. Br, bronchus; V, vessel; a, alveolus; Image are shown at 20X magnifications. E) Absolute cell count in BAL fluid. Absolute number of eosinophils (Eosino), macrophages (Macro), mononuclear agranulocytes (Mono), and neutrophils (Neutro) in BAL fluid was determined as described in Materials and Methods. Data were mean ± SEM. *P<0.05 vs. SHAM/PBS/VEH and ^†^P<0.05 vs. OVA/OVA/VEH; n = 5–6 mice in each group.

To determine the effects of baicalein (BAIC) on asthma features, male Balb/c mice were sensitized and challenged with OVA and treated with different concentrations of BAIC from the onset of allergen challenge (Fig. 1A). To determine the effect of baicalein on AHR, single chamber plethysmography was performed (see Materials and Methods). The concentration of methacholine (MCh) at which mice had developed 200% increase in enhanced pause (Penh) from the baseline values (MCh PC200 Penh) was determined. Penh is an index of airway hyperresponsiveness and it is directly proportional to airway obstruction. At the end of allergen challenge, the OVA/OVA/VEH group (allergic control mice) exhibited the development of AHR as those mice developed airway obstruction even at the low doses of methacholine than the SHAM/PBS/VEH (normal control mice). In this model, BAIC reduced AHR in a dose dependent manner and the optimal effective dose was 10 mg/kg (Fig. 1B). Therefore subsequent experiments were performed using the 10 mg/kg dose, unless otherwise indicated. For confirmation, we again estimated AHR, this time through invasive airway mechanics, which are considered more reliable. This confirmed that BAIC (10 mg/kg) treatment to OVA induced mice was associated with a significant reduction in AHR (Fig. 1C).

### BAIC Reduces Airway Inflammation and Airway Eosinophilia

To determine the effect of BAIC on airway inflammation, histopathological analysis was performed on sections stained with Haematoxylin and Eosin stained lung sections. As shown in Fig. 1D, the OVA/OVA/VEH mice developed perivascular and peribronchial infiltration of inflammatory cells including eosinophils, compared to the control (SHAM/PBS/VEH) mice. In contrast, the OVA/OVA/BAIC mice showed a significant reduction in inflammation both around the vessel and bronchi. This was confirmed by objective inflammation scoring (Table 1).

**Table 1:** Effects of Baicalein on IL-4, IL-13, IFN-γ, TLR-2, TLR-4, OVA specific IgE, IgG2a, eotaxin levels and airway inflammation score.

Groups	IL-4 (pg/100 µg protein)	IL-13 (pg/100 µg protein)	IFN-γ (A.U. pg/25 µg protein)	TLR-2 (pg/25 µg protein)	TLR-4 (pg/100 µg protein)	OVA Spec.IgE (A.U.)	OVA Spec.IgG2a (A.U.)	Eotaxin (pg/25 µg protein)	Inflammation score		
									PV	PB	Total
SHAM/PBS/VEH	52.7±7.9	11.8±1.1	18.2±0.3	17.2±7.3	3.8±2.2	0.6±0.1	0.9±0.5	23.2±2.5			
OVA/OVA/VEH	112.4±10.3*	47.5±2.6*	12.5±1.3*	59.9±7.1*	14.1±1.7	2.3±0.2*	1.8±0.2*	63.7±6.2*	3.6±0.08	2.9±0.1	6.5±0.2
OVA/OVA/BAIC	88.0±1.1^†^	34.7±1.5^†^	16.9±1.1^†^	16.2±6.8^†^	14.8±4.5	1.4±0.2^†^	2.2±0.1^†^	39.1±2.6^†^	1.9±0.1	1.2±0.1	3.1±0.2^†^

Data were mean ± SEM of three independent experiments.

* P<0.05 vs. SHAM/PBS/VEH group, and ^†^P<0.05 vs. OVA/OVA/VEH group.

Since eotaxin plays a significant role in the migration of eosinophils to the airway, we measured its levels in lung homogenates. As shown in Table 1, eotaxin levels were significantly higher in the OVA/OVA/VEH mice compared to the SHAM/PBS/VEH mice. BAIC treatment reduced the eotaxin levels significantly compared to the OVA/OVA/VEH mice. The increase in eotaxin levels was associated with an increase in the recruitment of eosinophils in the airway in the OVA/OVA/VEH mice (Fig. 1E) and baicalein treatment reduced airway eosinophilia.

To determine the effects of BAIC on Th_2_ cytokines and IgE, we measured the levels of IL-4 and IL-13 in lung tissue and OVA specific IgE in sera. As shown in Table 1, the levels of IL-4, IL-13 and OVA specific IgE were increased in the OVA/OVA/VEH mice compared to the SHAM/PBS/VEH mice. BAIC treatment was associated with a significant reduction of both the cytokines and also OVA specific IgE (Table 1). In addition, BAIC treatment significantly increased OVA specific IgG2a (Table 1). Since there was an increase in IgG2a, we measured IFN-γ levels in lung tissue. As shown in Table 1, IFN-γ levels were decreased in the OVA/OVA/VEH mice compared to the SHAM/PBS/VEH mice. However, BAIC treatment to allergic mice was associated with a significant increase in IFN-γ levels (Table 1).

Since baicalein appears to restore the balance the altered Th_1_/Th_2_ response in the allergically inflamed lungs, we tested the effect of baicalein on Toll-Like receptor proteins such as TLR-2 and TLR-4, as it is known that TLR-2 and TLR-4 skew the Th responses towards Th_2_ and Th_1_ respectively [19]. As shown in Table 1, both TLR-2 and TLR-4 were found to be increased in the OVA/OVA/VEH mice compared to the SHAM/PBS/VEH mice. BAIC treatment to allergic mice was associated with a significant decrease in TLR-2 levels, but TLR-4 levels were unchanged (Table 1).

### BAIC Reduces Airway Remodeling Changes

**Figure 2:**
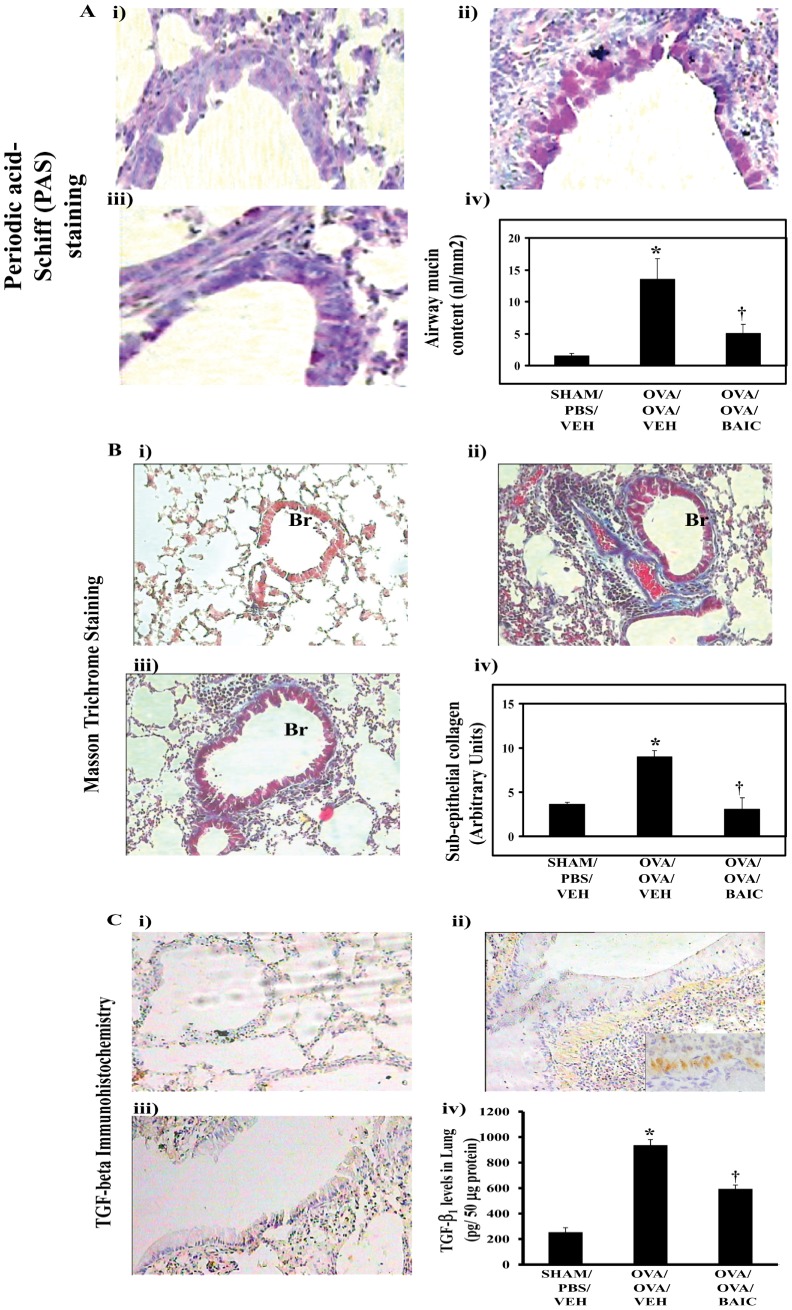
BAIC treatment reduced airway remodeling changes. A) Periodic acid staining was performed with lung tissue sections and airway mucin (iv) was estimated by quantitative morphometry. B) Masson Trichrome staining was performed with lung tissue sections and sub-epithelial collagen content (iv) was estimated by quantitative morphometry. C) IHC was performed in lung tissue sections to determine the expression of TGF-β_1_. Brown color indicates the positive expression of TGF-β_1_. C, iv) The levels TGF-β_1_ in lung tissue homogenates were determined by ELISA. All Images are at 20 X magnifications. Results were mean ± SEMs of three independent experiments. *P<0.05 vs. SHAM/PBS/VEH, ^†^P<0.05 vs. OVA/OVA/VEH group. Data were mean ± SEM of three independent experiments. *P<0.05 vs. SHAM/PBS/VEH, ^†^P<0.05 vs. OVA/OVA/VEH group. The diameters of bronchi which have been taken for morphometry analysis were 326.9±16.0 µm, 339.6±23.3 µm, and 326.4±47.3 µm for SHAM/PBS/VEH, OVA/OVA/VEH and OVA/OVA/BAIC, respectively.

To determine the effect of BAIC on airway remodeling changes, we determined the effect of BAIC on goblet cell metaplasia and sub-epithelial fibrosis. To determine the effect of BAIC on goblet metaplasia, we performed periodic acid-Schiff staining on the lung sections. As shown in Fig. 2A, the OVA/OVA/VEH mice displayed the increased goblet cell metaplasia and BAIC treatment reduced it, which was confirmed by morphometric analysis.

As shown in Fig. 2B, dense accumulation of collagen was found in sub-epithelial regions of bronchi and also around vascular regions in allergic control mice compared to normal control mice. Interestingly, BAIC treatment had significantly reduced the collagen deposition in the bronchovascular regions (Fig. 2B). This was further confirmed by morphometry. Since TGF-β_1_ is important in the development of sub-epithelial fibrosis, we determined the effect of BAIC on the levels and expression of TGF-β_1._ As shown in Fig. 2C, the OVA/OVA/VEH mice showed a significant expression of TGF-β_1_ especially in the subepithelial mesenchymal regions and increased levels in lung cytosol relative to the SHAM/PBS/VEH mice (Fig. 2C, iv). Interestingly, BAIC treatment resulted in the reduction in the expression and levels of TGF- β_1_ in lung (Fig. 2C, i–iv).

### Baicalein Treatment After the Development of Asthma Symptoms Reduces Airway Hyperresponsiveness and Airway Inflammation

**Figure 3:**
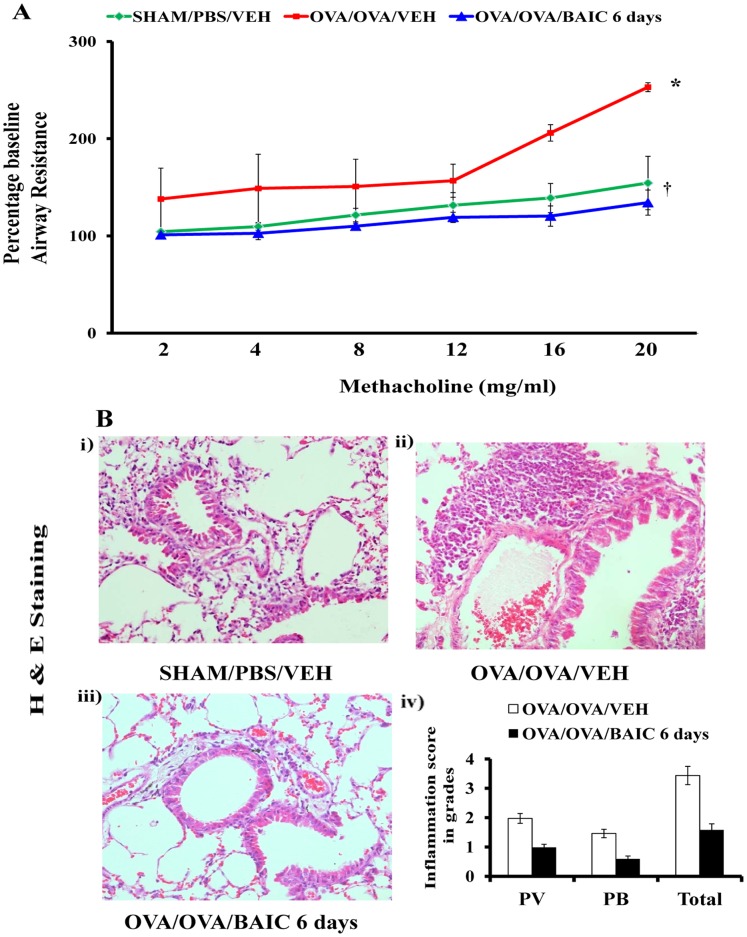
Therapeutic effect of Baicalein on AHR and airway inflammation. A) Male Balb/c mice were sensitized and challenged for 12 consecutive days as shown in Fig. 1A and OVA-induced mice were treated with baicalein from day 27 to 32 to see the therapeutic effect in mice which had developed asthma features. AHRs were determined on Day 33. A) Invasive airway mechanics were performed with increasing concentrations of methacholine aerosol and results were expressed as percentage baseline airway resistance assuming values derived from PBS aerosol was considered as baseline. B) Representative photomicrographs of Haematoxylin and Eosin staining were shown. Br, bronchus; V, vessel; a, alveolus; Image are shown at 20X magnifications. Inflammation score of the lungs was evaluated by experimentally blind experts and shown as perivascular (PV), peribronchial (PB) and Total (sum of both PV and PB). Data were the representative of three independent experiments. *P<0.05 vs. SHAM/PBS/VEH and ^†^P<0.05 vs. OVA/OVA/VEH; n = 5–6 mice in each group.

Since baicalein showed anti-asthma effect in the preventive model, we wanted to test its efficacy in a therapeutic model. To determine the therapeutic effect of baicalein in asthma, it was administered to the mice after the development of asthma symptoms (Fig. 1A). Since allergen exposure is likely to continue during asthma therapy of human subjects, OVA challenges were continued during the BAIC treatment, until day 32 (Fig. 1A). The OVA-sensitized and -challenged mice showed asthma features such as MCh induced AHR and airway inflammation after 6 days OVA challenge (data not shown). BAIC treatment was therefore started after 6 days of OVA challenge, but with the continuation of OVA challenge. In this therapeutic model, BAIC treatment reduced AHR as well as the infiltration of inflammatory cells in the bronchovascular regions and inflammation score (Fig. 3A, B).

### Baicalein Treatment Attenuates the AHR and Airway Inflammation Induced by IL-13 or IL-4

**Figure 4:**
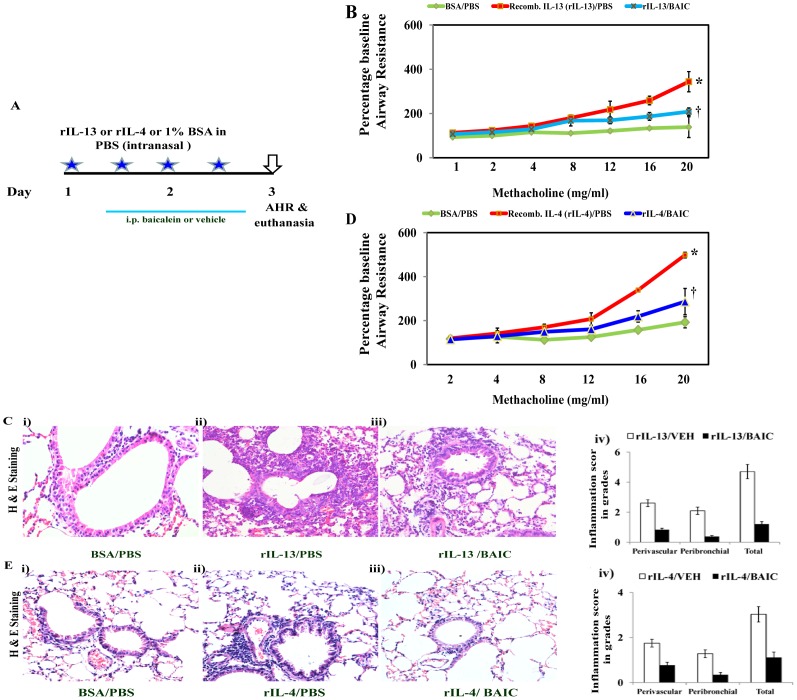
Effect of BAIC on AHR and airway inflammation induced by IL-13 or IL-4. A) Naïve Balb/c mice were anaesthetized with isoflurane and administered 4 doses of either vehicle (1% BSA in PBS) or recombinant IL-13 (rIL-13) or rIL-4 intranasally for two days and AHR was determined on 24 hrs after the last dose. Baicalein was administered intraperitoneally twice a day for 2 days. B, D) Percentage baseline airway resistance. C, E) Representative photomicrographs of Haematoxylin and Eosin staining were shown. Br, bronchus; V, vessel; a, alveolus; Images are shown at 20X magnifications. Inflammation score of the lungs. Data were the representative of 2–3 independent experiments. *P<0.05 vs. SHAM/PBS/VEH and ^†^P<0.05 vs. OVA/OVA/VEH; n = 5–6 mice in each group.

To further confirm the anti-asthma activities of BAIC, it was administered to naïve mice, which were given intranasal IL-13 or IL-4 (Fig. 4A). As expected, administration of IL-13 led to a significant increase in AHR and infiltration of inflammatory cells in the bronchovascular regions, with corresponding increase in inflammation score (Fig. 4B, C). However, BAIC administration to IL-13 treated mice led to a significant reduction in both AHR and airway inflammation (Fig. 4B, C). In addition, mice administered with IL-4 also showed an increase in AHR and airway inflammation, which was attenuated by BAIC (Fig. 4D, E).

### BAIC Reduces the Levels of 15-LOX Metabolites in Cytosol

**Figure 5:**
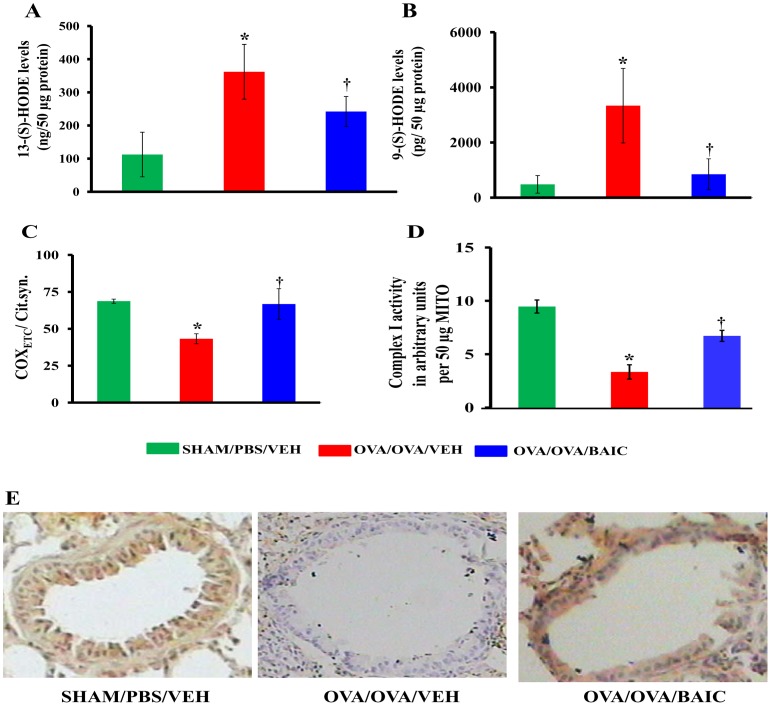
Baicalein reduces the levels of 15-LOX metabolites and restores mitochondrial function. A) 13-(S)-HODE, and B) 9-(S)-HODE were measured in lung cytosols. Mitochondria were isolated from fresh lung and activities of cytochrome c oxidase (C) and complex I (D) were estimated. E) Representative photomicrographs of immunohistochemically stained lung sections for subunit III of cytochrome c oxidase. Brown color indicates the positive expression. Data were mean ± SEM of three independent experiments. *P<0.05 vs. SHAM/PBS/VEH group, and ^†^P<0.05 vs. OVA/OVA/VEH group; n = 5–6 mice in each group.

Baicalein inhibits 15-LOX activity potently but not specifically [20], [21]. 15-LOX metabolites such as 13-S-HODE and 9-S-HODE are generated in high concentrations during mitochondrial degradation in the reticulocytes during the process of reticulocyte maturation [22], [23]. 15-LOX is also found to be increased in bronchial epithelia in human asthmatic conditions and mitochondrial dysfunction seems to be crucial in the pathogenesis of various respiratory diseases including asthma [23]. 15-LOX preferentially utilizes linoleic acid (LA) to produce 9 and 13-(S)-hydroxyoctadecaenoic acids (HODEs) and also minimally utilizes arachidonic acid (AA) to produce 12-(S)-hydroxyeicosatetraenoic acids (HETE) [23]. In this context, we determined the effect of baicalein on mitochondrial dysfunction and airway epithelial injury. We first measured the levels of 13-S-HODE and 9-S-HODE, which are the 15-LOX metabolites. As shown in Fig. 5A & B, the OVA/OVA/VEH mice had an increase in the levels of 13-S-HODE and 9-S-HODE compared to the SHAM/PBS/VEH mice. However, BAIC treatment significantly reduced the levels of both metabolites (Fig. 5 A, B).

### BAIC Increases the Activity of Cytochrome c oxidase and Restores the Reduction in the Expression of COX_ETC_ Subunit III

To determine the effect of baicalein on mitochondrial function in the experimental asthma model, we measured cytochrome c oxidase activity and normalized by respective citrate synthase activity in lung mitochondria. The results indicated that the normalized cytochrome c oxidase activity was decreased in the OVA/OVA/VEH mice compared to the SHAM/PBS/VEH (Fig. 5C). Also, there was a reduction in complex I activity in lung mitochondria of the OVA/OVA/VEH mice. In addition, subunit III was found to be predominantly expressed in bronchi of the SHAM/PBS/VEH mice, and was found to be significantly decreased in the OVA/OVA/VEH mice (Fig. 5D). The high expression of subunit III in bronchial epithelia may relate to vulnerability to inflammatory insults [24]. Interestingly, BAIC significantly restored the activities of cytochrome c oxidase and complex I with the restoration of the expression of subunit III of cytochrome c oxidase (Fig. 5C–E).

### BAIC Reduces the Cytochrome c Levels, Caspase 3 and Caspase 12 Activities in Lung Cytosol

**Figure 6:**
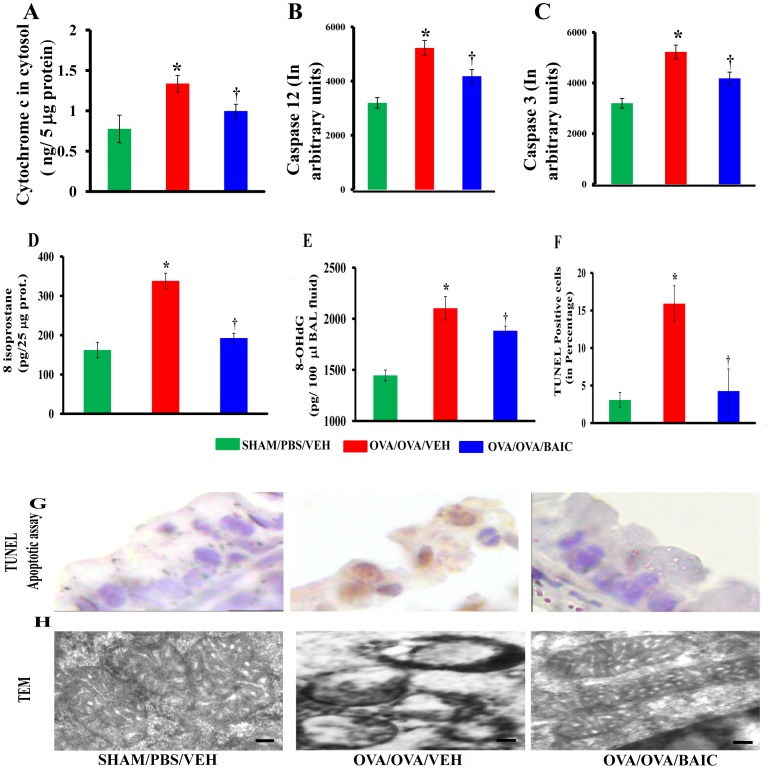
Baicalein reduced bronchial epithelial injury and restores mitochondrial ultrastructural changes in bronchial epithelia of asthmatic lungs. A) Lung cytosols were prepared. Cytochrome c (A), caspase 12 (B), Caspase 3 (C) and 8-isoprostane, marker of lipid oxidative stress (D), were estimated in lung cytosols. E) 8-hydroxy deoxyguanosine (8-OHdG), a marker of DNA oxidative stress, was estimated in BAL fluid supernatant. F–G) TUNEL apoptosis assay in lung tissue sections and apoptotic index to determine the number of TUNEL positive bronchial epithelia were estimated. Data were mean ± SEM of three independent experiments. *P<0.05 vs. SHAM/PBS/VEH, ^†^P<0.05 vs. OVA/OVA/VEH group. H) To determine the mitochondrial morphology with BAIC treatment, transmission electron microscopy (TEM) was performed with first generation bronchi. Fifty bronchial epithelial cells were visualized randomly and representative picture had been shown here from each group. Bars, 0.1 µm.

As BAIC restored the reduction in cytochrome c oxidase activity and complex I activity in allergic control mice, we further analyzed the cytochrome c levels and caspase 3 activities in lung cytosol. As shown in Fig. 6A, the OVA/OVA/VEH mice showed increased levels of cytochrome c in the lung cytosol compared to the SHAM/PBS/VEH mice. However, the OVA/OVA/BAIC mice showed a significant reduction of cytochrome c in the cytosol (Fig. 6A). In addition, the OVA/OVA/VEH mice also had increased activities of caspase 3 and caspase 12, treatment with BAIC was able to decrease their enzymatic activities. (Fig. 6B, C).

### BAIC Reduces Apoptosis of Bronchial Epithelia in Asthmatic Airway

We measured oxidative stress as a means of analyzing mitochondrial dysfunction using 8-isoprostane, a reliable marker of lipid peroxidation. As shown in Fig. 6D, 8-isoprostane was found to be significantly increased in the OVA/OVA/VEH mice compared to the SHAM/PBS/VEH mice, and was significantly reduced by BAIC.

Since increased cytochrome c stimulated the intrinsic apoptotic pathway, we measured the levels of 8-hydroxy-2′-deoxyguanosine (8-OHdG), a marker for the oxidative DNA damage, in BAL fluid supernatants. We found an increase in 8-OHdG levels in the OVA/OVA/VEH mice compared to the SHAM/PBS/VEH mice, and was reduced by BAIC treatment (Fig. 6E). Furthermore, we performed TUNEL apoptosis assay in lung sections to determine the apoptotic index in this organ. As shown in Fig. 6F & G, the OVA/OVA/VEH mice showed increased apoptosis, especially in bronchial epithelial cells. However, BAIC treatment showed a significant reduction in DNA fragmentation (Fig. 6F & G).

### BAIC Restores Mitochondrial Ultrastructural Changes

To determine the effect of BAIC on morphological changes of the mitochondria in bronchial epithelium, we performed transmission electron microscopy. As shown in Fig. 6H, the OVA/OVA/VEH mice showed maximal mitochondrial damage in the form of loss or disruption of cristae, swelling and thinned outer membrane. However, BAIC treatment significantly restored the mitochondria to its normal architecture (Fig. 6H).

### BAIC Reduces IL-13 Mediated Mitochondrial Dysfunction and Airway Injury

**Figure 7:**
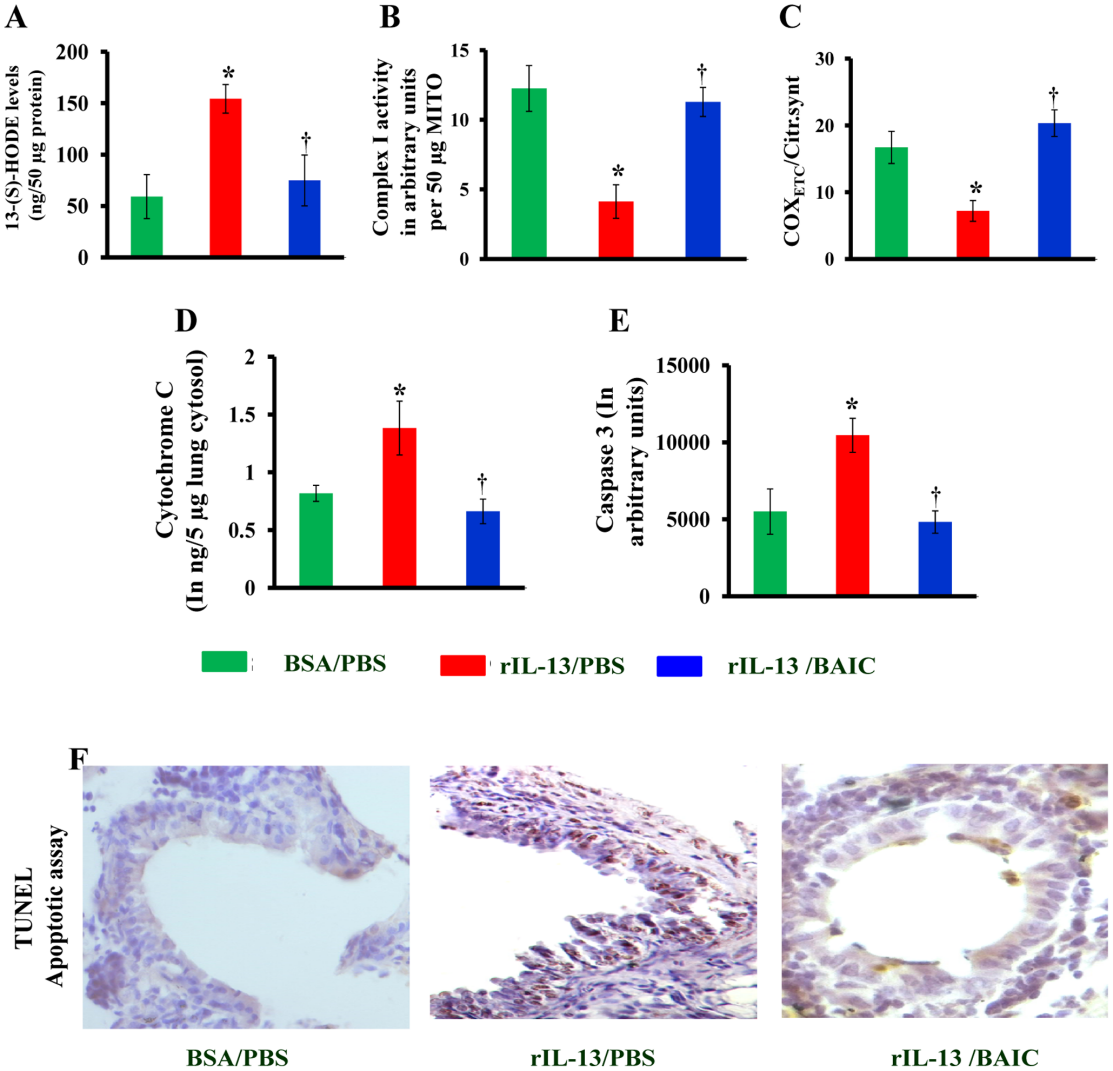
Baicalein reduces IL-13 induced mitochondrial dysfunction and bronchial epithelial injury. A) Mitochondria and cytosols were isolated from fresh lungs. 13-S-HODE levels in lung cytosol (A), activities of complex I (B) and cytochrome c oxidase (C), cytochrome c (D) and caspase 3 (E) were estimated. F) TUNEL apoptosis assay in lung tissue sections. Brown color indicates the TUNEL positive apoptotic cells. Data were mean ± SEM of three independent experiments. *P<0.05 vs. BSA/PBS group, and ^†^P<0.05 vs. rIL-13/PBS group; n = 5–6 mice in each group. Table 1. Effects of Baicalein on IL-4, IL-13, IFN-γ, TLR-2, TLR-4, OVA specific IgE, IgG2a, eotaxin levels and airway inflammation score.

To further determine the effect of baicalein on airway injury, we determined the features of mitochondrial dysfunction and TUNEL apoptotic assay in lung sections from IL-13 treated mice which were treated with BAIC. As shown in Figure 7A, BAIC treatment reduced the IL-13 mediated increase in 13-S-HODE levels in lungs. This BAIC mediated reduction in 13-S-HODE levels was associated with the restoration of mitochondrial function which is demonstrated by an increase in complex I activity, cytochrome c oxidase activity in lung mitochondria and reduction in cytochrome c in lung cytosol (Fig. 7B–D). In addition, BAIC treatment reduced the caspase 3 activity and apoptosis of bronchial epithelia induced with IL-13 administration (Fig. 7E–F).

## Discussion

Earlier it was believed that airway inflammation plays the dominant role in asthma pathogenesis and airway epithelial injury was believed to be the downstream product of airway immune cell infiltration. Thus, therapeutic strategies were focused on reducing airway inflammation. However, recent reports including ours suggest that mitochondrial dysfunction and epithelial injury are crucial in allergic airway inflammatory diseases, such as asthma [3]–[12]. In addition, it has been demonstrated that epithelia may influence the immune status of the lung. It has also been strongly suggested that different therapeutic approaches can be devised by focusing more on protecting vulnerable airways against allergen induced injury [8]. In this study, we have evaluated the potency of baicalein in reducing airway injury, mitochondrial dysfunction and alleviating the features of allergic asthma. We used allergic models and Th_2_ cytokine models to demonstrate the efficacy of baicalein in preventing airway injury. We found that baicalein ameliorates mitochondrial dysfunction, reduces airway injury and attenuates various features of airway inflammation, not only in the allergic model but also the Th_2_ cytokine models.

We found that baicalein reduced the perivascular and peribronchial infiltration of inflammatory cells, BAL fluid eosinophilia, eotaxin in lung and OVA specific IgE levels in sera. Earlier studies showed that baicalein reduced the production of eotaxin and stromal derived factor [14], [25], which are well known for their chemoattractant properties to recruit different types of leukocytes and eosinophil from the vasculature. Interestingly, baicalein inhibits TNF-α induced adhesion molecule expression in cultured human umbilical vein endothelial cells [26]. In this study, baicalein treatment to allergic mice modulated the Th_1_/Th_2_ response by increasing IFN-γ, OVA specific IgG2a and decreasing IL-4 and IL-13. Further, baicalein treatment to OVA induced mice resulted in the reduction in TLR2 levels without affecting TLR-4 levels. A similar reduction of TLR-2 by baicalein has been reported in neurons [27]. In addition, baicalein reduced the levels of 15-LOX metabolites such as 13-(S)-HODE and 9-(S)-HODE, which have been shown to be important in various features of inflammation such as chemotactic stimulation of leukocytes, and the activation and migration of lymphocytes [28]. Consequently, the modulation of these different proinflammatory mediators by baicalein might explain the reduction of airway inflammation we observed in this study.

The reduction of 13-S-HODE and 9-S-HODE are interesting in asthmatic lungs since these metabolites are produced in large quantities by reticulocytes during the process of mitochondrial degeneration. Evidently, baicalein treatment is associated with the restoration of mitochondrial functions with increase in the activities of cytochrome c oxidase and complex I, and mitochondrial ultrastructural changes in asthmatic bronchial epithelia. Therefore, the reduction of mitochondrial dysfunction and restoration of ultra structural changes by baicalein could either be due to 15-LOX inhibition, or by indirect mechanisms such as the reduction of IL-4, IL-13 and lipid peroxidation. However, since baicalein is a nonspecific 12/15-LOX inhibitor, the observed effects of baicalein may not be due to 15-LOX inhibition alone. We have also found that baicalein treatment reduced the levels of secretory phospholipase A2 (data not shown), which is known to be a proinflammatory mediator in asthma pathogenesis [29]. Whether this is a direct or indirect effect remains to be studied later.

Mitochondrial damage is commonly associated with epithelial injury in respiratory diseases [30]. We found that baicalein reduced DNA fragmentation in bronchial epithelial cells and oxidative DNA damage. The anti-apoptotic effect of baicalein in OVA induced asthma model is interesting. It is known that baicalein causes apoptosis in various tumor cells [31]–[33]. In contrast, anti-apoptotic effects of baicalein have been demonstrated on normal cells under various stress conditions [34]–[37]. It is well known that cancer cells evade mitochondrial apoptotic mechanisms through depletion of mitochondria and shifting to a non-mitochondrial metabolism (Warburg effect). We speculate that this apparent anomaly may relate to such differences. However, further investigations are required to determine the effects of baicalein on primary lung epithelial cells.

Epithelial injury is one of the primary pathogenic mechanisms in asthma, since it initiates airway remodeling by inducing various growth factors such as TGF-β_1_. It has been shown that baicalein has anti-fibrotic properties in *in vitro* studies [38]. In this study, baicalein had reduced the expression of TGF-β_1_ along with the reduction of collagen deposition in sub-epithelial regions. This is likely related to the inhibition of epithelial injury and pro-survival effects discussed above. Interestingly, baicalein treatment also reduced goblet cell metaplasia, which is an important aspect of other obstructive airway diseases like chronic bronchitis (Fig. 2). The mechanism of this is unclear, but appears to be a direct effect since baicalein pretreatment to IL-13 induced human bronchial epithelia reduced Muc5Ac levels in an *in vitro* study (Muc5Ac levels in pg/25 µg protein were 6.2±0.4, 11.5±1.3, 11.6±7.2, 6.7±1.6, 5.7±1.9 for uninduced, IL-13 induced with vehicle treated, and IL-13 induced and pretreated with 7.5 µM, 15 µM and 30 µM baicalein, respectively). 15-LOX has been previously implicated in goblet cell metaplasia [39]–[40]. Since baicalein has a broad spectrum of biological activities, the exact mechanism underlying its anti-asthma activities in this study are not clear. However, the inhibition of various proinflammatory mediators such as IL-4, IL-13, TGF-β_1_, 15-LOX, and reduction of 15-LOX metabolites such as 13-(S)-HODE and 9-(S)-HODE, are likely to lead to reduction of bronchial epithelial injury and consequent airway remodeling could be possible mechanisms by which baicalein reduces asthma features.

In summary, baicalein treatment to either allergic or Th_2_ cytokine induced mice reduces airway epithelial injury, ameliorates mitochondrial dysfunction, and various features of airway inflammation, in a mouse models and this could have an important implication in developing therapeutic strategies towards airway injury in asthma.

## Materials and Methods

### Animals

This animal study was performed in strict accordance with the guidelines provided by Committee for the Purpose of Control and Supervision of Experiments on Animals (CPCSEA). All animals (8–10 weeks old male BALB/c mice were obtained from National Institute of Nutrition, Hyderabad, India or Central Drug Research Institute, Lucknow and maintained in CSIR-Institute of Genomics and Integrative Biology, Delhi) utilized in this study were under protocols approved by Institutional Animal Ethics Committee of CSIR-Institute of Genomics and Integrative Biology, Delhi, India. All the surgical procedures were performed under sodium pentobarbital anesthesia and maximum efforts were taken for minimum suffering of animals.

### Grouping, Sensitization, Challenge and Treatment of Mice

There were two experimental models: a) OVA model and b) Th_2_ cytokine models. In OVA model, there were two sets of experiments: 1) Dose titration experiments in which there were 6 groups of mice (n = 5–6 in each): SHAM/PBS/VEH (normal controls, VEH-vehicle), OVA/OVA/VEH (OVA controls, OVA, chicken egg ovalbumin, Grade V, Sigma) and OVA/OVA/BAIC 0.1, 1, and 10 (0.1 mg/kg, 1 mg/kg, and 10 mg/kg baicalein, respectively, Cayman); and 2) In verification experiments, there were two models: a) preventive model and b) therapeutic model. In preventive model, there were 3 groups (n = 6 in each): SHAM/PBS/VEH, OVA/OVA/VEH and OVA/OVA/BAIC (baicalein, 10 mg/kg). In therapeutic model, there were 3 groups (n = 6 in each): SHAM/PBS/VEH, OVA/OVA/VEH and OVA/OVA/BAIC 6 days (baicalein, 10 mg/kg). In both models, mice were sensitized on days 0, 7, and 14 with 50 µg OVA adsorbed in 4 mg alum or 4 mg alum alone and were challenged from Day 21 to Day 32 with 3% OVA or PBS consecutively as described earlier [10]. Baicalein was dissolved in 5–10% dimethyl sulfoxide, vehicle. BAIC or VEH was administered intraperitoneally (i.p.) from day 21 to 32 twice a day in 100 µl volume per dose in preventive model and similar dose was given from day 27 to 32 in therapeutic model (Figure 1A). It is to be noted that other studies have used very high doses of baicalein (up to 520 mg/kg oral) without having any adverse effects [41], [42]. We have used two different treatment durations to demonstrate the preventive and therapeutic effects of baicalein. Importantly we have not observed any adverse effects such as inflammation, loss of weight etc. in these mice or in naïve mice which have been given similar doses of baicalein for the similar duration (data not shown).

In Th_2_ cytokines model, 3 µg (30 µl) carrier-free recombinant murine IL-13 or IL-4 (R&D Systems) in 1% BSA or 1% BSA alone was instilled into the nasal openings of each isoflurane-anesthetized mouse, twice a day for 2 days and mice were treated with BAIC or VEH intraperitoneally (i.p.) twice a day for two days (Figure 4A). AHR measurement and euthanasia were performed on Day 3.

### Measurement of Airway Hyperresponsiveness

Airway hyperresponsiveness (AHR) to methacholine (MCh) was determined on Day 33 by single chamber body plethysmography or invasive instrument as described earlier [10], [43], [44]. Briefly, each mouse was taken into the Buxco single chamber, acclimatized for 5–10 minutes, baseline recordings were measured for 5 minutes. Then mice were challenged with PBS or different concentrations of MCh aerosol and signal were recorded with in-built software (Buxco) to determine the Penh values which are reliable in Balb/c mice [10]. Then MCh PC200 [the partial concentration of methacholine which is required to double the baseline enhanced pause (Penh)] was calculated. To confirm the findings from non-invasive body plethysmography, respiratory mechanics were determined during mechanical ventilation (flexiVent system, Scireq, Canada) as previously described [44]. Briefly, anesthetized mice were intubated after tracheostomy, ventilated with computer-controlled ventilator and airway resistance with different concentrations of MCh was estimated using the flexiVent system (Scireq) that integrates ventilator with the respiratory mechanics [44].

### Transmission Electron Microscopy (TEM) and Lung Histopathology

TEM with first generation bronchi was performed [10]. Briefly, all lungs were similarly fixed using combined in situ whole body perfusion and immersion [10]. Fixed lungs were dissected using a dissection microscope to select the first generation bronchi. Slices from these bronchi were further processed, stained and viewed with TEM. In other experiments, Hematoxylin & Eosin staining, Periodic acid staining and Masson trichrome staining were performed in another portion of lung to assess inflammatory changes, goblet cell metaplasia and sub-epithelial fibrosis, respectively. Airway mucin content and collagen deposition were determined by quantitative morphometry as described earlier [10], [45] with little modifications.

### Isolation of Whole Lung Mitochondria and Cytosolic Separation

After sacrifice and bronchoalveolar lavage (BAL) [10], mitochondria isolation and cytosolic separation were performed [10] from lung.

### Measurements of 13-(S)-hydroxyoctadecaenoic Acid (HODE), 9-(S)-HODE in Cytosolic Fractions

Lung cytosols were used for ELISA of 9-(S)-HODE (Oxford Biomedical research) and 13-(S)-HODE (Assay Designs Inc.) as per the manufacturer’s instructions.

### Measurements of Interleukin-4, IL-13, IFN-γ, TLR-2, TLR-4, Eotaxin, TGF-β_1_ and OVA Specific IgE, and IgG2a

Lung tissue homogenates were used for sandwich ELISA (BD Biosciences for IL-4, IFN-γ and TGF-β_1_, R&D systems for IL-13 and eotaxin and USCN life Science for TLR-2 and TLR-4) and results were expressed in picograms and normalized by protein concentrations [10]. OVA specific IgE & IgG2a were measured as described earlier [10].

### Measurements of 8-isoprostane and 8-OHdG

8-isoprostane, a marker of lipid peroxidation, was measured in lung homogenates by competitive ELISA and results were expressed in pg/25 µg protein. 8-OHdG, a marker of oxidative DNA damage, was measured in BAL fluid supernatants by competitive ELISA and results were expressed in pg/100 µl BAL fluid supernatants.

### Measurements of Total Cytochrome C Oxidase Activity, Complex I Activity, Activities of Caspase 3 & Caspase 12 and Cytochrome c in Lung

Total cytochrome c oxidase activity in lung mitochondria, caspase 3 activity and cytochrome c levels in lung cytosols were performed [10]. Complex I activity (MitoSciences, USA) and caspase 12 activity (Biovision, USA) were determined as per manufacturer’s instructions.

### Immunohistochemistry

This was performed as described earlier [10] with commercial goat polyclonal antibodies for cytochrome c oxidase III and TGF-β_1_ (Santa Cruz Biotechnology, Inc.) as primary antibodies and HRP conjugated anti-goat IgG as a secondary antibody. Negative control experiments were performed by using either gamma globulin as isotype controls (Jackson Immunoresearch Laboratories, Inc.) or omission of primary antibodies.

### Terminal Deoxynucleotidyl Transferase dUTP Nick End Labeling (TUNEL) Apoptotic Assay

TUNEL assay of apoptosis was performed on lung tissue sections using an in situ apoptosis detection kit (Dead End Calorimetric TUNEL system; Promega) as described earlier [46]–[48]. Bronchial epithelial cells per lung section were examined by experimentally blind investigators and the numbers of apoptotic cells are expressed as a percentage of the whole epithelial cell population (apoptotic index).

### In Vitro Culture of Human Bronchial Epithelia and Muc5ac ELISA

To determine the direct effect of baicalein on mucin expression, BEAS-2B cells, human bronchial epithelia, were pretreated with vehicle (DMSO) or different concentrations of baicalein (7.5 µM, 15 µM and 30 µM) 3 hrs before induction without or with 20 ng/ml IL-13 for 48 hrs. After 48 hrs of IL-13 induction, cells were harvested and the levels of Muc5ac ELISA were determined in cell lysates (USCN life Science, China).

### Statistical Analysis

Data are expressed as mean ± SEM. Unpaired student’s ‘t’ test and ANOVA with post-hoc correction were performed to calculate the statistical significance which was set at p≤0.05.
